# The Impact of Rare Human Variants on Barrier-To-Auto-Integration Factor 1 (Banf1) Structure and Function

**DOI:** 10.3389/fcell.2021.775441

**Published:** 2021-11-08

**Authors:** Maddison Rose, Bond Bai, Ming Tang, Chee Man Cheong, Sam Beard, Joshua T. Burgess, Mark N. Adams, Kenneth J. O’Byrne, Derek J. Richard, Neha S. Gandhi, Emma Bolderson

**Affiliations:** ^1^ Queensland University of Technology (QUT), Cancer and Ageing Research Program, Centre for Genomics and Personalised Health, Translational Research Institute (TRI), Brisbane, QLD, Australia; ^2^ Princess Alexandra Hospital, Woolloongabba, QLD, Australia; ^3^ School of Chemistry and Physics, Queensland University of Technology, Brisbane, QLD, Australia

**Keywords:** nuclear envelope, DNA binding, nuclear integrity, BANF1, human variants

## Abstract

Barrier-to-Autointegration Factor 1 (Banf1/BAF) is a critical component of the nuclear envelope and is involved in the maintenance of chromatin structure and genome stability. Banf1 is a small DNA binding protein that is conserved amongst multicellular eukaryotes. Banf1 functions as a dimer, and binds non-specifically to the phosphate backbone of DNA, compacting the DNA in a looping process. The loss of Banf1 results in loss of nuclear envelope integrity and aberrant chromatin organisation. Significantly, mutations in Banf1 are associated with the severe premature ageing syndrome, Néstor–Guillermo Progeria Syndrome. Previously, rare human variants of Banf1 have been identified, however the impact of these variants on Banf1 function has not been explored. Here, using in silico modelling, biophysical and cell-based approaches, we investigate the effect of rare human variants on Banf1 structure and function. We show that these variants do not significantly alter the secondary structure of Banf1, but several single amino acid variants in the N- and C-terminus of Banf1 impact upon the DNA binding ability of Banf1, without altering Banf1 localisation or nuclear integrity. The functional characterisation of these variants provides further insight into Banf1 structure and function and may aid future studies examining the potential impact of Banf1 function on nuclear structure and human health.

## Introduction

The separation of the genome from the cytoplasm is a defining characteristic of eukaryotic cells (Alvarado-Kristensson and Rossello, 2019). For many years the nuclear envelope was viewed as a physical barrier to protect the cellular genetic material from damage and degradation. However, more recently, studies have highlighted its crucial role in gene regulation and other important cellular processes. Supporting this, defects in the nuclear envelope and associated proteins have also been linked with several human diseases, including premature ageing syndromes (Worman et al., 2010). Barrier-to-Autointegration Factor (Banf1/BAF) is a small non-specific DNA binding protein, conserved amongst multicellular eukaryotes. Banf1 was initially identified for its capacity to inhibit autointegration of retroviruses, such as HIV, into their genome (Chen and Engelman, 1998). Banf1, functions as a dimer and binds to the phosphate backbone of the DNA, compacting the DNA in a looping process (Segura-Totten et al., 2002). The ability of Banf1 to bridge distant DNA sites has been shown to be required for the correct assembly of the nucleus (Samwer et al., 2017). In unperturbed cells, Banf1 is localised to the nuclear envelope and binds to several critical components of the chromatin and nuclear envelope, including histones, Lamin A and Emerin (Lee et al., 2001; Bengtsson and Wilson, 2006; Cai et al., 2007; Montes de Oca et al., 2009). Several nuclear envelope proteins, such as LEMD2 and Emerin contain Lem domains, which have been identified as sites of Banf1-binding (Laguri et al., 2001; Shumaker et al., 2001). Highlighing its importance in maintaining nuclear morphology, loss of Banf1 results in a loss of nuclear envelope integrity and aberrant chromatin organisation. Our previous work has also characterised the role of Banf1 in regulating the activity of DNA repair proteins, including Poly (ADP-ribose) polymerase 1 (PARP1) and DNA-dependent Protein kinase (DNA-PK) (Bolderson et al., 2019; Burgess et al., 2021).

A single point-mutation in the N-terminal domain of Banf1 is associated with the severe premature aging syndrome, Néstor–Guillermo Progeria Syndrome (NGPS) (Cabanillas et al., 2011; Puente et al., 2011; Paquet et al., 2014). Premature ageing is intrinsically linked with genome stability pathways (Gonzalo and Kreienkamp, 2015; Burla et al., 2018). Consistent with this, cells from NGPS patients exhibit defective PARP1 activity and impaired repair of oxidative lesions, supporting a model whereby Banf1 is crucial to reset oxidative-stress-induced PARP1 activity (Bolderson et al., 2019).

Banf1 has also been shown to have a key role in ensuring appropriate disassembly and reassembly of the nuclear envelope during mitosis, predominately regulated by Banf1’s phosphorylation state (Gorjanacz, 2013; Molitor and Traktman, 2014). Phosphorylated Banf1 cannot bind to Lem domain proteins and DNA; therefore, promoting the dissociation of chromosomes from the nuclear envelope during prophase (Nichols et al., 2006). Similarly, once cell division has occurred, Banf1 dephosphorylation is essential for reformation of the nuclear envelope (Gorjanacz, 2013; Zhuang et al., 2014).

More recently, cytosolic Banf1 has been shown to relocalise to nuclear envelope rupture sites due to Banf1’s affinity for double-stranded DNA (dsDNA), allowing for the recruitment of rupture repair proteins (Halfmann et al., 2019). Furthermore, Banf1’s affinity for dsDNA outcompetes that of cyclic guanosine monophosphate–adenosine monophosphate synthase (cGAS), a dsDNA sensor that is essential for activation of stimulator of interferon genes (STING) (Ma et al., 2020; Wan et al., 2020). Therefore, Banf1 is essential to protect self dsDNA from cGAS in the event of a nuclear rupture (Li et al., 2013; Guey et al., 2020).

Given the importance of Banf1 in maintaining cellular homeostasis, it is crucial we increase our understanding of the association between Banf1 human variants and their structure and function in human cells. Previously, a study investigated several Banf1 variants identified from the Exome Aggregation Consortium (ExAC) cohort of 60,706 unrelated individuals and speculated that several Banf1 variants might impact the dsDNA binding affinity (Lek et al., 2016; Dharmaraj et al., 2019). Here, we extend this previous study and investigate the effect of rare human variants on Banf1 structure and function, using molecular modelling, biophysical and cell-based analyses. Specifically, our results confirm that some variants impact the affinity of Banf1 DNA binding without altering Banf1 localisation or nuclear integrity.

## Materials and Methods

### GnomAD Database Searches

The gnomAD v2.1.1 and ExAC servers were queried for ‘*Banf1’ via* the gnomAD browser (Karczewski et al., 2020). Missense Banf1 mutations were identified from these servers, and several variants - that were common across both servers were selected for further analysis.

### Chemical Reagents

All chemical reagents were purchased from Sigma, unless otherwise stated.

### Cell Lines

U-2OS cells were obtained from CellBank Australia (Cat # 92022711) and grown in RPMI media, supplemented with 10% foetal bovine serum. Cells were maintained at 37°C, at atmospheric oxygen and 5% CO_2._

### Antibodies

The antibodies used were as follows: anti-Flag M2 (F3165, Sigma-Aldrich at 1:500 for immunofluorescence (IF) and 1:1,000 for western blotting (WB), anti-Emerin (5430S, Cell Signalling Technology at 1:500 for IF), anti-Actin Ab-5 (612,656, Bioscience International at 1:3,000 for WB). For IF, secondary antibodies; Alexa Fluor 488 (A32766, Molecular Probes, 1:300) and 594 (A32754, Molecular Probes, 1:300). Secondary antibodies used for WB were Donkey anti-Mouse 800 nm (IRDye 800CW 926-32212, LiCor, 1:5,000), Donkey anti-Rabbit (IRDye 680LT 926-28023, LiCor, 1:5,000).

### Banf1 Expression Constructs

The Flag-Banf1 construct was synthesised by Genscript in the pcDNA3.1+N-DYK vector in the BamHI-XhoI cloning sites and the variants were created *via* site-directed mutagenesis (Genscript) (Bolderson et al., 2019). These constructs were sequenced using the CMV primer (5′- CGC​AAA​TGG​GCG​GTA​GGC​GTG -3′). The His-Banf1 was synthesised by Genscript in the pET-28a (+) vector in the NdeI-XhoI cloning sites and the variants created *via* site-directed mutagenesis (Genscript). These constructs were sequenced using the T7 primer (5′- TAA​TAC​GAC​TCA​CTA​TAG​G-3′) (Bolderson et al., 2019).

### Expression of Flag-Banf1 Variants

U-2OS cells were transfected with the above Banf1 expression constructs using Fugene HD transfection reagent (Promega) as per the manufacturer’s guidelines. Transfection was confirmed at 48 h post-transfection by western blot with anti-FLAG antibodies.

### Immunoblotting

Cells were lysed (lysis buffer: 20 mM HEPES pH 7.5, 250 mM KCl, 5% glycerol, 10 mM MgCl_2_, 0.5% Triton X-100, protease and phosphatase inhibitor cocktail (Thermofisher Scientific) at 48 h post-transfection. Cells were sonicated and lysates were cleared by centrifugation. 20 µg of protein lysates were separated on a 4–12% SDS-PAGE gel (Invitrogen), prior to blocking in Intercept Blocking Buffer (LiCor Bioscience) and immunoblotting with anti-Flag and anti-Actin Ab-5 antibodies. Immunoblots were imaged using an Odyssey imaging system (LiCor).

### Immunofluorescence

Immunofluorescence was performed as previously (Bolderson et al., 2019). Briefly, U-2OS cells were seeded 24 h post-transfection in a 96 well plate and allowed to adhere for 24 h. Cells were pre-treated with extraction buffer for 5 min to visualise chromatin bound protein (Bolderson et al., 2010), prior to fixation in 4% PFA. Cells were permeabilised for 5 min in 0.2% Triton X-100 prior to blocking for 30 min in 3% bovine serum albumin. Cells were incubated in anti-Flag and anti-Emerin primary antibodies for 1 h at room temperature, prior to incubation in Alexa-conjugated secondary antibodies for 1 h at room temperature. Cells were countered stained in Hoechst 3,342 (1:1,000). Cells were imaged on a DeltaVision pDV deconvolution microscope with 100x/1.42 Oil objective (Applied Precision, Inc). ImageJ was utilised to assemble immunofluorescence images.

### Nuclear Envelope Quantification

Immunofluorescent staining and imaging were completed as prior described. 200 cells per Banf1 variant were manually determined to have Flag-Banf1 localised/not localised to the nuclear envelope for each biological replicate. Values were normalised to the proportion of Flag wild-type (WT) Banf1 cells with Banf1 localised to the nuclear envelope. Emerin staining was utilised as a control to visualise the nuclear envelope.

### Nuclear Roundness

Immunofluorescent staining and imaging were completed as prior described. For each biological replicate, 200 cells per Banf1 variant were manually determined to have normal/abnormal nuclear roundness. Values were normalised to the proportion of Flag WT Banf1 cells with normal nuclear roundness. Emerin staining was utilised as a control to visualise the nuclear envelope.

### Banf1 Purification

Purification of recombinant proteins was adapted from (Bolderson et al., 2019). Plasmids expressing HexaHis-tagged Banf1 WT or mutants (Genescript) were transformed into BL21 (DE3) pLysS *E. coli*. Cells were grown at 37°C in 500 ml LB media (Luria-Bertani medium, 10 g/L Tryptone, 10 g/L NaCl, 5 g/L Yearst Extract, pH 7.0) and protein expression was autoinduced with 0.6% (w/v) glycerol, 0.05% (w/v) glucose, 0.625% lactose (w/v). *E. coli* were harvested 20 h after autoinduction by centrifugation and stored overnight at -80°C. Cell pellet was lysed in 25 ml of lysis buffer (25 mM HEPES pH 7.5, 150 mM NaCl, 0.01% IGEPAL) and sonicated. Cell lysates were centrifuged for 30 min at 40,000 *g* and the supernatants discarded. The insoluble inclusion bodies containing HexaHis Banf1 were solubilised in buffer (25 mM HEPES pH 7.5, 150 mM NaCl, 0.01% IGEPAL, 25 mM imidazole) supplemented with 6 M guanidine hydrochloride, and kept for 90 min at 4°C under agitation. The lysate was then centrifuged and the clarified supernatant incubated with HIS-Select^®^ Nickel Affinity Gel for 90 min at 4°C under agitation. The affinity gel was washed extensively with solubilisation buffer and incubated in 10 mM ATP, 5 mM MgCl_2_ for 20 min at 4°C. The protein was eluted from the beads in buffer K (20 mM KH_2_PO_4_ pH 7.5, 0.5 mM EDTA, 10% glycerol) supplemented with 300 mM KCl and 250 mM imidazole. Eluents were supplemented with 100 mM DTT and incubated overnight at 4°C to reduce any remaining disulphide bonds.

Purified proteins were concentrated on 10 kDa Amicon^®^ Ultra-4 centrifugal filter unit (Millipore) to a volume of 250 µl or until precipitate is visible and loaded onto a Superose 6 10/300 GL size exclusion chromatography column (GE Healthcare) and run with buffer K containing 300 mM KCl. Fractions containing monomeric and dimeric Banf1 were pooled, concentrated, and stored at −80°C.

### DNA Probe Labeling and Purification

All oligonucleotides were purchased from Integrated DNA Technology (IDT), forward: 5′Cy5-AGGAGCGCCAGACCCACCAAGAGCCCTCTATCGGTTGGGA, reverse: 5′-TCC​CAA​CCG​ATA​GAG​GGC​TCT​TGG​TGG​GTC​TGG​CGC​TCC​T. All oligonucleotides were purified on 12% polyacrylamide 8 M urea gels prior to use. Equal molar of corresponding oligonucleotides were mixed in annealing buffer (10 mM Tris pH 7.5, 50 mM NaCl, 1 mM MgCl_2_), incubated in a 100°C water bath then slowly cooled to room temperature. Annealed oligos were purified on 8% polyacrylamide gel in 1x TBE buffer and concentrated. The concentration was determined using OD_260_ and the extinction coefficient of the oligonucleotide (ε_Cy5=_665,449 L/mol.cm).

### Electrophoretic Mobility Shift Assay

Reactions were carried out in 10 µl of buffer (10 mM KH_2_PO_4_, 100 mM NaCl, 0.01% IGEPAL) containing 5 nM of Cy5-labelled DNA probe. Proteins and DNA probe were incubated for 30 min at 37°C. Reactions were resolved on 8% polyacrylamide gel in 1x TBE buffer run at 4°C for 90 min at 80 V. Gels were scanned using a Typhoon FLA 9000 scanner and quantified using Image Studio Lite Ver 5.2.

### Prediction of Effect of Mutations on Banf1-DNA Interactions

The 3D structure of Banf1 dimer with dsDNA (pdb code: 2BZF; 7 nucleotide; biological assembly (Bradley et al., 2005)) was used to predict folding free energy changes caused by Banf1 mutations. We predicted the affinity change (ΔΔG) using the mutation Cutoff Scanning Matrix (mCSM) server (http://biosig.unimelb.edu.au/mcsm/protein_dna) (Pires et al., 2014; Pires and Ascher, 2017; Zhang et al., 2018), mCSM-NA (http://biosig.unimelb.edu.au/mcsm_na/) (Pires and Ascher, 2017) and PremPDI (https://lilab.jysw.suda.edu.cn/research/PremPDI/) (Zhang et al., 2018). These servers have been widely used to predict protein-nucleic acids binding affinities (Pires et al., 2014).

### CD Spectroscopy

CD spectroscopy was carried out using the JASCO J-1500 circular dichroism spectrophotometer. Protein samples were buffer exchanged to a 10 mM potassium phosphate, 50 mM sodium fluoride buffer (10 mM K_2_HPO_4_, 50 mM NaF) with a Zeba Spin Desalting Column (Cat # 89882, Thermo Scientific) to avoid interference with the CD signal. All CD signals were corrected to a blank value based on the CD trace of the buffer used. For each experiment, 200 μl at 0.2 mg/ml of purified proteins were used. Temperature gradient CD measurements were performed with a point CD measurement every 0.1°C with three technical repeats. In addition, a full CD trace from 185–260 nm was also taken every 5°C increase from 20 to 90°C with three technical repeats each, with the temperature increasing by 1°C per minute. All CD measurements were performed at a scanning speed of 100 nm/min, and 1 nm bandwidth. During CD spectrophotometer experiments, N_2_ gas was used and regulated to provide a 7 l pm flow rate throughout, system was flushed with N_2_ gas 15 min before and 15 min after usage.

All CD spectra data was analysed using software provided with the JASCO J-1500 instrument. The overlaying of the CD spectra of each sample was done using the Spectra Analysis tool, and the thermal stability was calculated using the Thermal Denaturation Multi-Analysis tool within the JASCO Spectra Manager software.

## Results

### Identification of Rare Banf1 Human Variants

**FIGURE 1:**
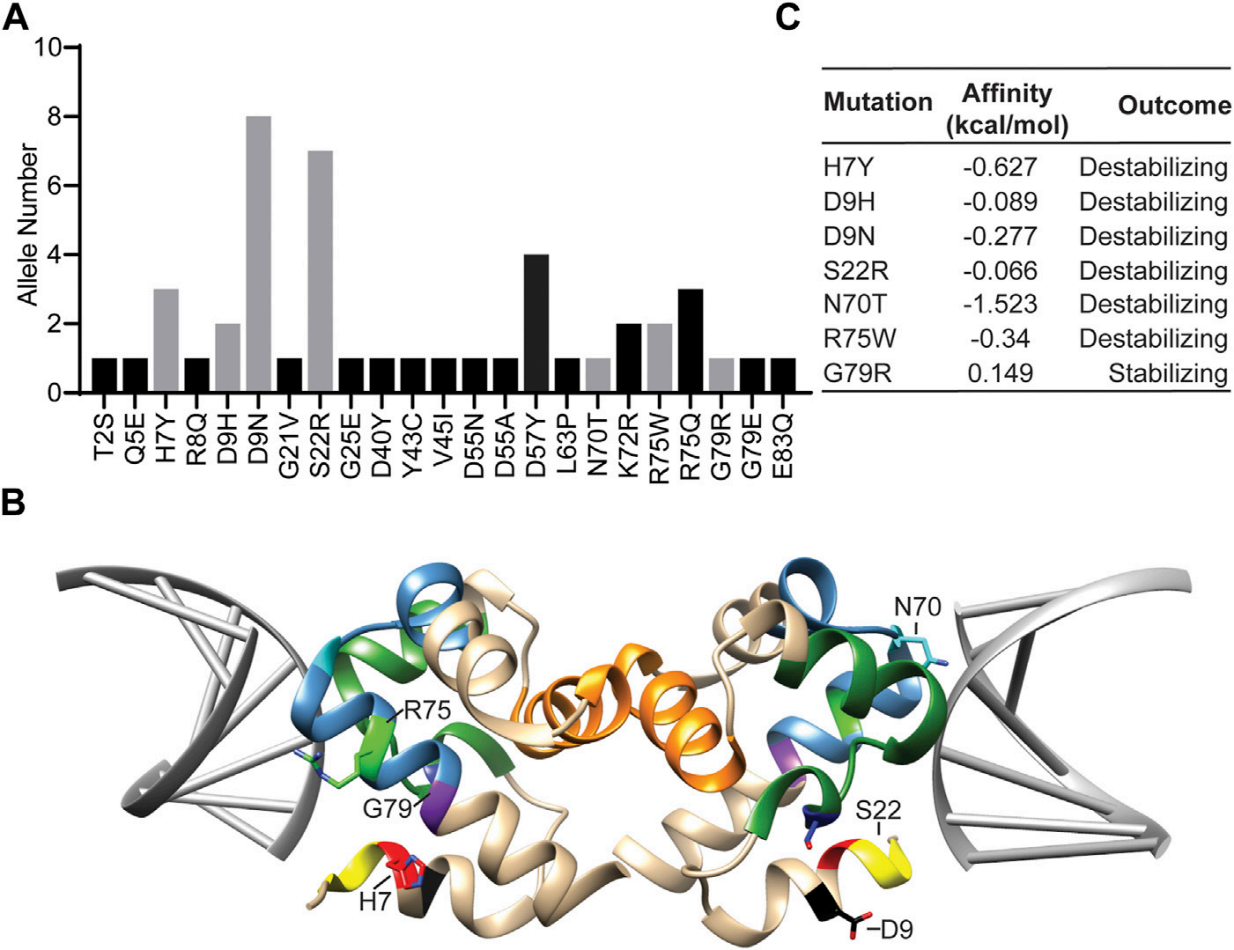
Rare human Banf1 variants identified from the gnomAD server show altered predicted DNA binding. **(A)** The Banf1 variants that were identified from the GnomAD server, with the allele numbers shown. The grey bars represent the variants selected for this study. **(B)** Ribbon representation of the molecular structure of Banf1 dimer (coloured in tan) in complex with DNA double helix coloured in grey (PDB id: 2BZF). H7 is highlighted in red, D9 in black, S22 in yellow, N70 in blue, R75 in green and G79 in purple. Sidechains of some of the highlighted residues are only displayed in one of the Banf1 monomers. **(C)** Predicted alterations in Banf1 DNA binding affinity (ΔΔG (kcal/mol) generated by the mCSM server. Negative ΔΔG (kcal/mol) are defined as destabilising and positive ΔΔG (kcal/mol) are defined as stabilising the Banf1:DNA interaction.

In order to identify rare Banf1 human variants, we first utilised the Genome Aggregation database (gnomAD) v3.1 short variant data set, containing 76,156 genomes from unrelated individuals. In addition to the 13 missense variants previously identified using the ExAC server (Dharmaraj et al., 2019), this analysis found an additional 10 Banf1 variants (Figure 1A). From these 23 Banf1 human variants we selected 7 variants that were present on both the ExAC server (Dharmaraj et al., 2019) and the GnomAD server (Figures 1A,B). These mutants include H7Y, N70T, D9N, D9H, S22R, R75W and G79R, which are mapped on the dimer structure of Banf1-DNA complex (Pdb code; 2BZF (Bradley et al., 2005)) as highlighted in Figure 1B. Among the 7 Banf1 mutations examined, R75W and N70T are within the DNA-binding region of the pseudo helix-hairpin-helix region (in steel blue), and H7Y is adjacent to the N-terminal DNA binding region (in yellow), suggesting that these mutations may affect the binding of Banf1 to DNA. It should be noted that there are no matched data available to determine whether these Banf1 variants are likely to be pathogenic.

In order to investigate the impact of Banf1 protein variants on the structure and function of Banf1, we used the mCSM, mCSM-NA and PremPDI servers (Pires et al., 2014) to explore the impact of all of these mutants on the binding capacity of Banf1 to DNA (Figure 1C, Supplementary Figure S1). Here, we found that the servers predicted that all the mutants have some degree of impact upon Banf1-DNA binding affinity, with the majority predicted to destabilise Banf1 structure. It should, however, be noted that the server predictions were not in complete agreement for all of the variants, we will discuss the potential reasons for this later in this manuscript. In particular, the mCSM server showed that R75W, H7Y, N70T, D9N, D9H and S22R were predicted to destabilise the Banf1 interaction with DNA and G79R was predicted to have a stabilising effect (Figure 1C). We considered that the difference in DNA binding capacity between the Banf1 variants and wild-type (WT) Banf1 may occur due to several reasons, including the changes in the protein structure (protein secondary structure) or the interactions between DNA and residues in the vicinity of mutation sites. Motivated by these predictions, we aimed to experimentally explore if these human variant missense mutations; 1) alter the secondary structure of Banf1 protein, 2) impact the DNA binding capacity of Banf1 in EMSA assays, 3) impact Banf1 cellular functions including cellular localisation and nuclear integrity.

### The Effects of Variants on Banf1 Structure

**FIGURE 2:**
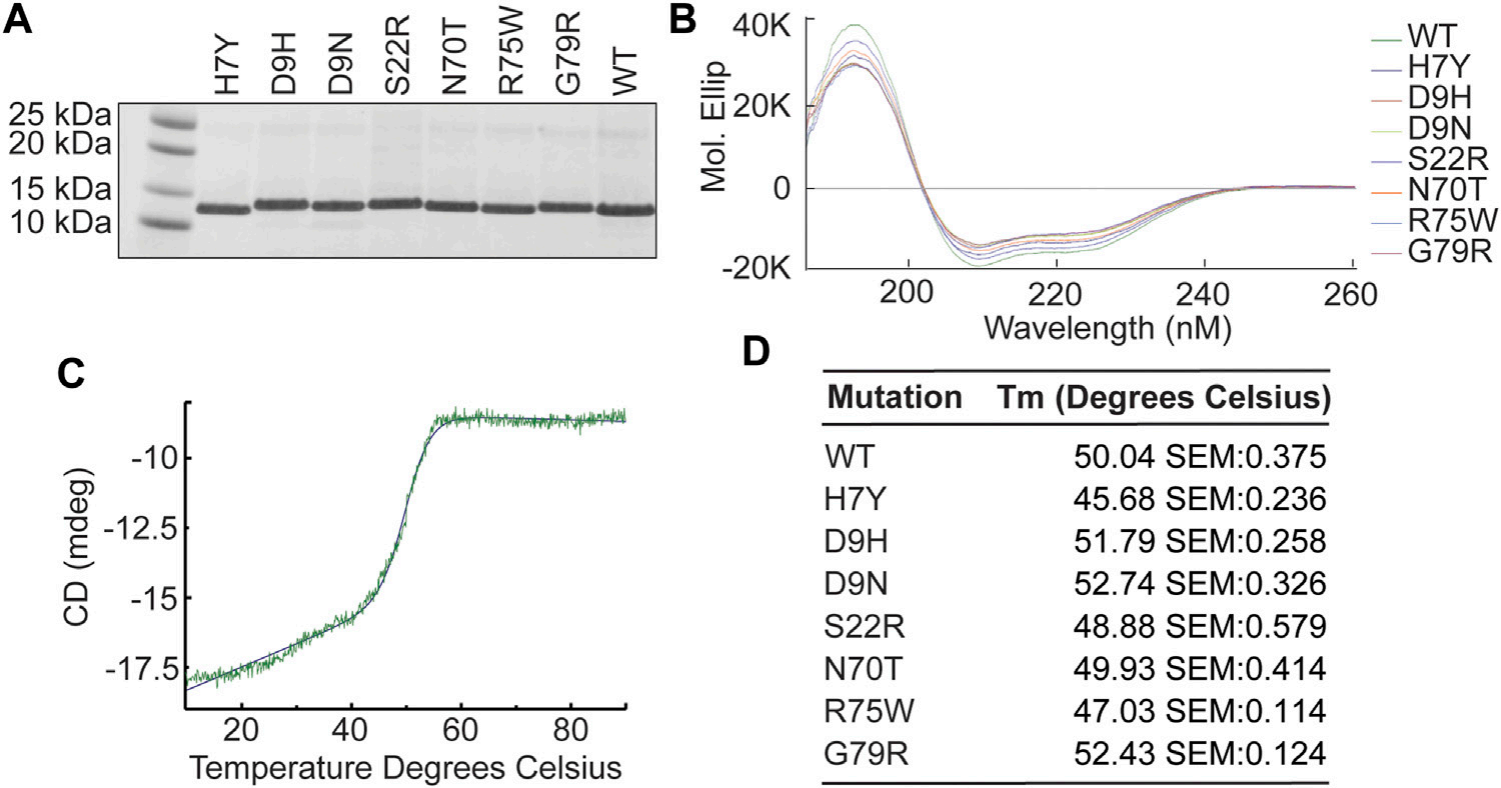
Banf1 variants do not significantly alter Banf1 secondary structure. **(A)** Banf1 His-tagged WT and variant proteins were expressed and purified from *E.coli* cells. Purified proteins were run on an SDS PAGE gel and stained with Coomassie. **(B)** Representative molecular ellipticity trace over a wavelength of 185–260 nm (n = 3). The molecular ellipticity values were calculated based the molar concentration of each protein sample and the CD values, which were obtained using JASCO J-1500 circular dichroism spectrophotometer. **(C)** Representative curve fitting of thermal stability analysis of point CD measurements at 222 nm over a temperature gradient from 10 to 90°C, taken in three technical repeats every 0.1°C. **(D)** Thermal disassociation temperature of Banf1 wild-type, H7Y, D9H, D9N, S22R, N70T, R75W, and G79R variants.

Circular dichroism spectroscopy (CD spec) experiments were carried out for each indicated variant, as well as WT Banf1 to determine impact of mutations on the protein secondary structure. Recombinant Banf1 WT and variant proteins were expressed and purified from *E. coli* (Figure 2A). At room temperature, all variants were confirmed to have a typical α-helical profile (Micsonai et al., 2015) with no significant changes in secondary structure when compared with WT Banf1 protein (Figure 2B). To investigate whether the variants were stabilising or destabilising the secondary structure of Banf1, temperature gradient experiments were also carried out using CD spec. A sigmoidal curve was fitted to each of the variants’ temperature gradient traces at 222 nm (Figure 2C) to determine the effect of temperature on the unfolding of the variants compared to WT Banf1 (Figures 2C,D). This demonstrated that the H7Y, S22R, and R75W variants disrupt the thermal stability of Banf1 (Figure 2D).

### Effects of Variants on Banf1 DNA Binding Ability

**FIGURE 3:**
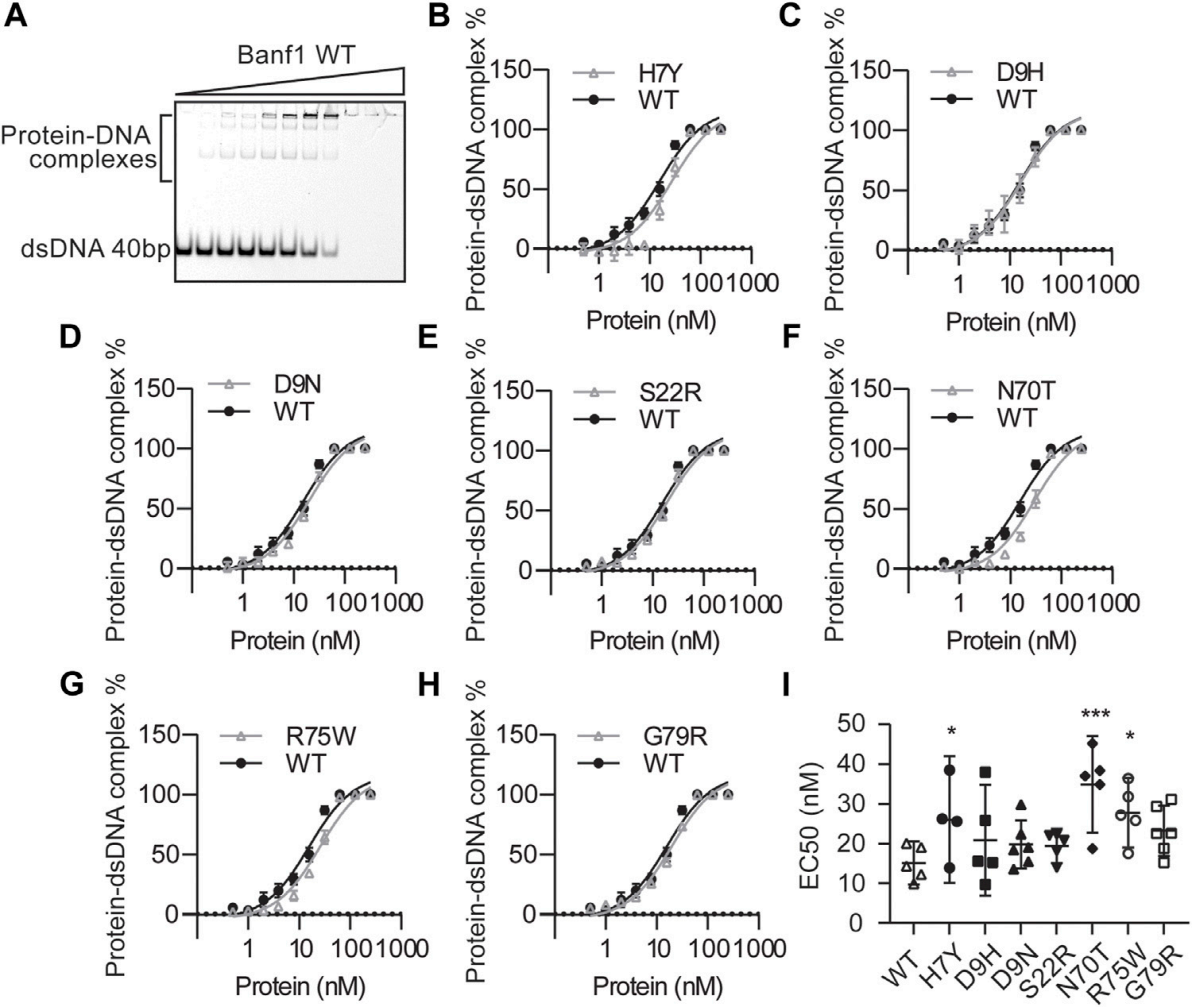
Banf1 variants display differing dsDNA binding affinities. **(A)** Representative electrophoresis mobility shift assay (EMSA) of Banf1 WT (0.5, 1.0, 2.0, 3.9, 7.8, 15.6, 31.3, 62.5, 125, 250 nM) binding to dsDNA of 40bp labelled with 5′ Cy5. EMSA comparing percentage of Banf1 WT with Banf1 variant **(B)** H7Y*,* **(C)** D9H, **(D)** D9N, **(E)** S22R, **(F)** N70T, **(G)** R75W, **(H)** G79R binding to dsDNA. Curves were fitted with non-linear regression model and plotted with a logarithmic *x*-axis. **(I)** EC50 quantification of Banf1 variant-dsDNA binding derived from least four independent experiments. Error bars denote 95% confidence intervals as determined by the binding curve fitting is shown. *p < 0.05; **p < 0.01, ***p < 0.005.

Next, we examined the effect of the Banf1 variants on the DNA binding ability of Banf1 using electrophoresis mobility shift assays (EMSAs). Increasing amounts of Banf1 protein were titrated into Cy5-labelled 40 oligonucleotide double-stranded DNA. Interaction between Banf1 and DNA was observed as retardation in the migration of the complex across polyacrylamide gel (Figure 3A) with an EC_50_ of 15.12 ± 1.959 nM. Analysis of the Banf1 variants (Figures 3B–I, Supplementary Figure S2), showed that H7Y, N70T, and R75W demonstrated a significantly weaker binding ability to DNA compared with WT Banf1 (Figures 3A,B,F,G) with a significantly increased EC_50_ of 26.06 ± 5.018 nM, 34.87 ± 4.379 nM and 27.76 ± 3.166 nM respectively (Figure 3I). The weaker DNA binding of H7Y and R75W correlates with the predicted destabilisation in the Banf1 variants from the mCSM server (Figure 1C) and the disruption of thermal stability (Figure 2D). The weaker DNA binding of N70T also correlates with the predicted destabilisation, although its thermal stability was not affected (Figure 1C).

### The Effects of Variants on Banf1 Cellular Function

**FIGURE 4:**
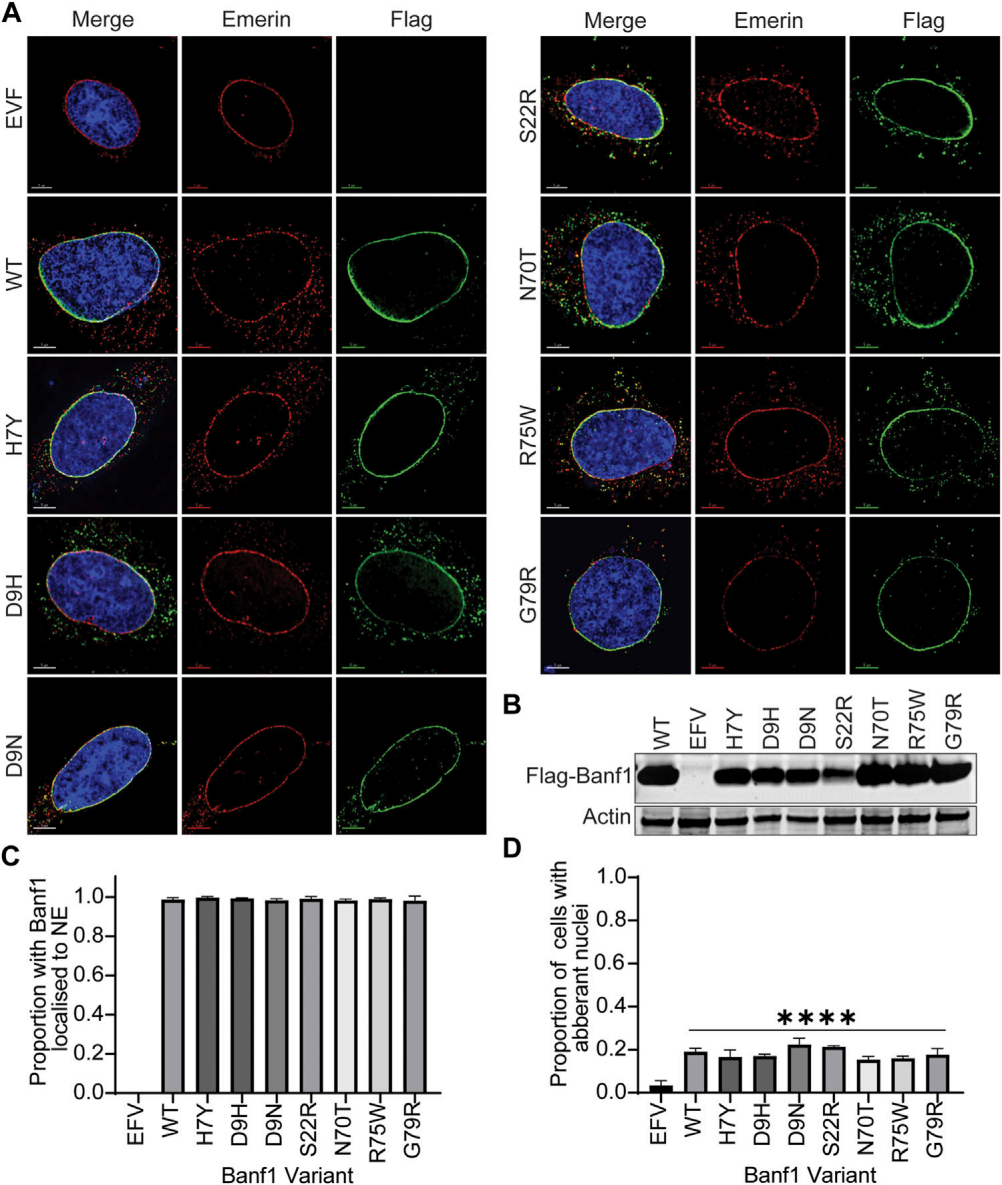
Banf1 variants localise to the nuclear envelope and do not affect nuclear morphology. **(A)** Representative immunofluorescent microscopy images of U-2OS cells transfected with EFV, Banf1 WT and Banf1 variants. Cells were stained with anti-Flag (Green) antibody and anti-Emerin (Red) to visualise the nuclear envelope. Cells were counterstained with Hoechst 3,342 (blue). **(B)** Representative immunoblot showing Flag-Banf1 WT and Banf1 variant expression in U-2OS cells. Anti-actin was utilised as a loading control. **(C)** Quantification of the proportion of Banf1 WT and variant transfected U-2OS cells with Banf1 localised to the nuclear envelope. **(D)** Quantification of aberrant nuclei in with EFV, Banf1 WT and Banf1 variant transfected U-2OS cells. Quantifications are based on 200 cells/condition in at least three independent experiments. Error bars denote standard deviation of the mean. EFV: empty Flag vector control. ***p < 0.001.

To explore the effects of Banf1 mutation on its cellular function, we expressed Flag-tagged -WT and variant Banf1 proteins in human cells. Given the role of Banf1 in nuclear envelope formation, we investigated if the Banf1 variants had an impact on the localisation of Banf1 to the nuclear envelope and, subsequently the nuclear morphology. To assess this, immunofluorescent microscopy was performed on U-2OS cells expressing Flag-Banf1 WT or human variants (Figures 4A,B). Using an antibody against Emerin, to visualise the nuclear envelope, it was determined that the specific point mutations investigated did not significantly alter the localisation of Banf1, with nuclear envelope localisation observed that was comparable to that of the Flag WT transfected cells (Figure 4C). Given that it has been previously shown that exogenous expression of Nestor-Guillermo progeria syndrome-associated Banf1 A12T mutation induces aberrations in nuclear morphology (Paquet et al., 2014), we next investigated if a similar phenotype was induced by the Banf1 variants selected for this study. Our findings demonstrated that transfection with the Flag-Banf1 variants did not significantly increase the number of cells with aberrant nuclear morphology compared to U-2OS cells transfected with WT Flag-Banf1 (Figure 4D). Overexpression of WT Banf1 also significantly increased the proportion of cells with aberrant nuclear morphology, which is consistent with our prior findings (Paquet et al., 2014).

## Discussion

Nuclear envelope proteins have an important role in critical cellular functions, including the correct breakdown and reassembly of the nuclear envelope following mitosis (Gorjanacz, 2013; Molitor and Traktman, 2014). Highlighting the importance of nuclear envelope proteins, mutations in genes encoding several of these proteins are associated with human diseases including Emerin (Emery-Dreifuss Muscular Dystrophy), Lamin A/C (Hutchinson-Gilford progeria syndrome) and Banf1 (Nestor-Guillermo progeria syndrome) (Worman et al., 2010). The atomic structure of Banf1 as an obligate dimer has been solved, including its two DNA binding sites (Bradley et al., 2005; Cai et al., 2007). Several *in vitro* studies have also characterised Banf1 residues responsible for DNA and histone binding (Umland et al., 2000; Segura-Totten et al., 2002; Montes de Oca et al., 2009). Here, we have investigated the effect of known Banf1 human variants on Banf1 structure and function.

We observed that while none of the coding amino acid variants caused a significant change in the secondary structure of Banf1, three of the variants did alter the ability of Banf1 to bind DNA. Banf1 R75W, H7Y and N70T were shown to have a destabilising effect on the DNA binding of Banf1, as predicted by the mCSM server. The CD spec analysis of these variants, provided a potential reason for the decrease in DNA binding ability of R75W and H7Y, showing a significant decrease in the thermal stability of Banf1 protein with these mutations. Significantly, supporting that these residues are involved in DNA binding, the R75, H7 and N70 amino acids are located on the surface of Banf1 involved in binding of Banf1 to DNA (Umland et al., 2000). Specifically, the Banf1 K6 residue, proximal to H7, has been shown to be involved in Banf1 binding to DNA previously, implicating this region in DNA binding and perhaps providing an explanation for a decrease in DNA binding of the H7Y mutant (Umland et al., 2000; Segura-Totten et al., 2002). R75 has also been implicated in Banf1 DNA binding previously, with mutation to R75E completely abrogating DNA binding (Umland et al., 2000; Segura-Totten et al., 2002). Supporting this, we observed that the R75W variant led to a significant decrease in DNA binding, similar to the R75E mutant (Umland et al., 2000; Segura-Totten et al., 2002). N70 is within 3.5Å away from DNA in the co-crystalised Banf1-DNA complex, suggesting that this region of Banf1 is likely involved in DNA binding, supporting the decreased DNA binding by the N70T variant we observed (Umland et al., 2000).

The comparison of Banf1-DNA binding predictions between different servers suggested that the utility of computational stability predictors may be inconsistent for predicting the stability of protein and interactions between BANF1 protein and DNA in the presence of mutations/variants, except for the effect of N70T and R75W (Supplementary Figure S1). We suggest that this could be largely due to alternate molecular mechanisms other than the protein destabilisation underlying many pathogenic mutations. We also consider that the performance of the servers could be influenced by the quality of the crystal structure submitted and that the algorithms optimise the side chain configurations, but do not take into account the effects produced by backbone conformational movements (Pires et al., 2014; Pires and Ascher, 2017; Zhang et al., 2018). Also of consideration is that Banf1 is reported to bind to non-specific dsDNA of different sizes and in our computational analysis, we have used Banf1 structure in conjunction with a short 7 nucleotides DNA molecule, due to the availability of Banf1:DNA crystal structures. We consider it likely that in cells, Banf1 might interact with a heterogeneous mixture of dsDNA and form high-order complexes, which creates additional complexities in predicting its DNA binding ability in such circumstances and may explain the disparity between the predicted and observed Banf1 DNA binding abilities.

Banf1 has been characterised as a nuclear envelope protein, required to maintain normal nuclear morphology (Furukawa, 1999; Segura-Totten and Wilson, 2004). Similarly to overexpression of WT Banf1, all of the human variants we have examined here were shown to localise to the nuclear envelope in human cells and had a similar effect to WT Banf1 on nuclear morphology. This perhaps suggests that Banf1 DNA binding activity is not required for the localisation of Banf1 to the nuclear envelope and that disruption of nuclear morphology is not due to changes in DNA binding ability. We acknowledge that the disruption of the DNA binding may not be sufficiently decreased in these variants to cause a phenotypic effect. However, the disruption of DNA binding in the R75W, H7Y and N70T variants was similar to our previous observations with the A12T mutant, which causes a similar change in DNA binding and a change in cell morphology when expressed (Paquet et al., 2014). This observation supports the hypothesis that a slight decrease in the binding ability of Banf1 is unlikely to be the cause of the changes in cell morphology and that these changes in phenotype might be attributed to altered binding to other protein interactors or another function of Banf1. However, further investigation into Banf1 structure and function is required to confirm this. We can also not exclude that in the current study the endogenous expression of wild type Banf1 may be sufficient to stabilise binding of the variant proteins to the nuclear envelope. It should be noted that the individuals carrying Banf1 variants are all heterozygous, with the exception of D9H, which is homozygous in one individual, so the presence of the endogenous Banf1 wild type protein could be considered to more closely resemble what is occurring in the cells of people carrying the variant proteins.

In summary, we have examined the effect of 7 rare Banf1 human variants on Banf1 structure and function, including identifying three variants that decrease DNA binding ability *in vitro*. Further investigation of these variants in terms of their binding to other protein partners, such as Lamin A and histones, their impact on cell division and DNA repair processes will shed further light on the role of Banf1 in cells and its impact on human health.

## Data Availability

The raw data supporting the conclusions of this article will be made available by the authors, without undue reservation.
