# Supplementary material for: The Impact of Rare Human Variants on Barrier-To-Auto-Integration Factor 1 (Banf1) Structure and Function
Source: Front Cell Dev Biol. 2021 Nov 8;9:775441. doi: 10.3389/fcell.2021.775441 (PMC8606531; doi:10.3389/fcell.2021.775441)
Supplement: Supplementary file 1 [file DataSheet1.PDF]

	mCSM server*	mCSM-NA*		PremPDI**
**Mutation**	Predicted affinity change (kcal/mol)	Stability effect (kcal/mol)	Predicted affinity change (kcal/mol)	ΔΔG (kcal/mol)
**H7Y**	-0.627 (destabilising)	0.077 (stabilising)	0.193	-0.08
**D9H**	-0.089 (destabilising)	-0.927 (destabilising)	1.544	-0.63
**D9N**	-0.277 (destabilising)	-0.895 (destabilising)	0.129	-0.39
**S22R**	-0.0666 (destabilising)	-0.45 (destabilising)	1.337	-0.18
**N70T**	-1.523 (destabilising)	-0.324 (destabilising)	0.929	1.14 (deleterious)
**R75W**	-0.34 (destabilising)	-0.785 (destabilising)	-0.466 (reduced affinity)	0.2
**G79R**	0.149 (stabilising)	-1.373 (destabilising)	0.504	0.25

Supplementary Figure 1. Predicted alterations in Banf1 DNA binding affinity (ΔΔG (kcal/mol). Predictions were generated by the mCSM, mCSM-NA and PremPDI servers.

*mCSM and mCSM-NA servers- predicted affinity change: A negative value corresponds to a mutation predicted as reducing affinity; while a positive value corresponds to a mutation predicted as increasing affinity.

**PremPDI server-A positive and negative signs correspond to destabilizing and stabilizing mutations predicted to decrease and increase binding affinity correspondingly. PremPDI server classifies a mutation as deleterious if ΔΔG is higher or equal to 1.10 kcal mol-1.

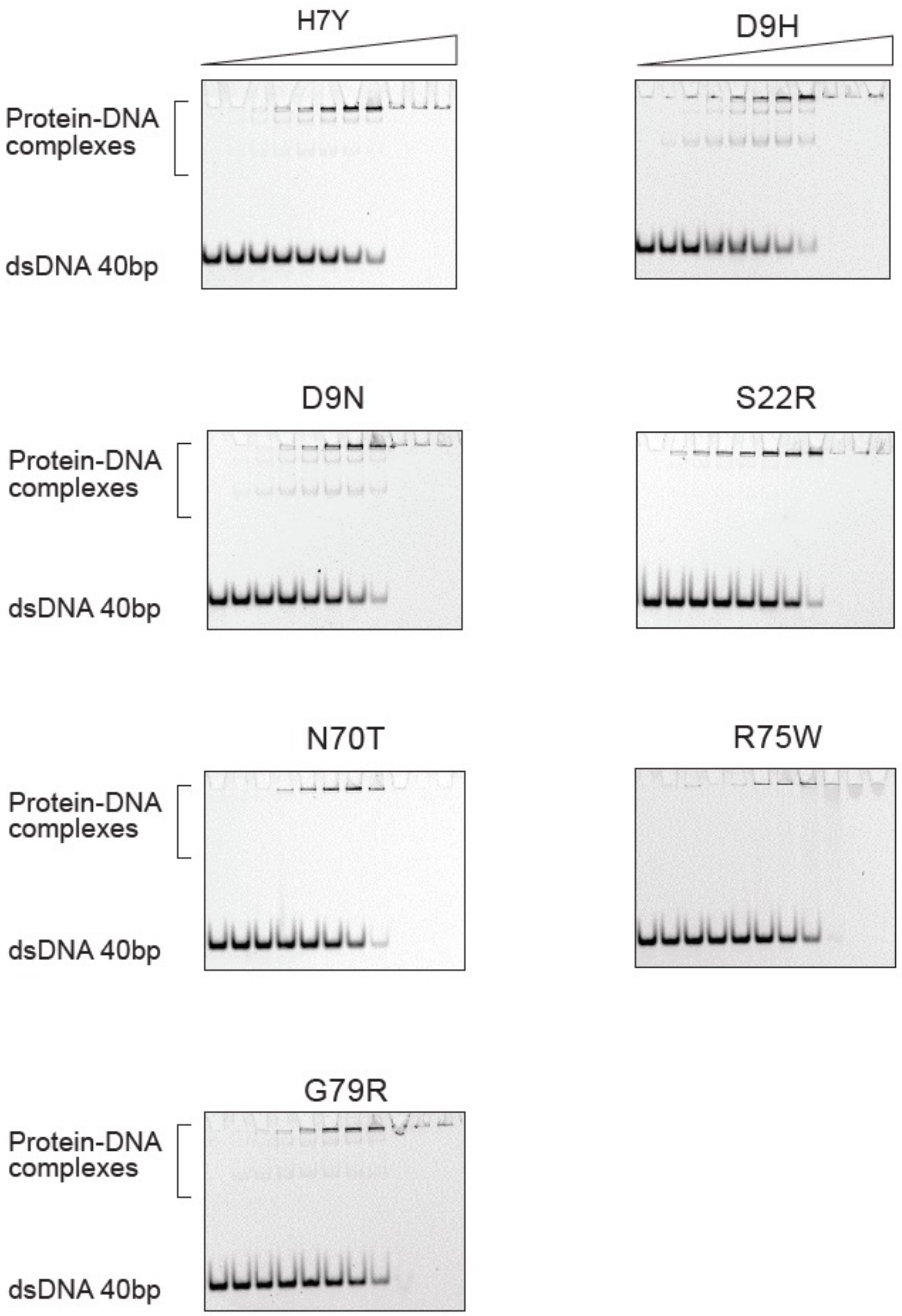

Supplementary Figure 2. Banf1 variants display differing dsDNA binding affinities. Representative Electrophoresis Mobility Shift Assays (EMSA) of Banf1 WT (0.5, 1.0, 2.0, 3.9, 7.8, 15.6, 31.3, 62.5, 125, 250 nM) binding to dsDNA of 40bp labelled with 5’ Cy5. EMSA comparing percentage of Banf1 WT with Banf1 variant **(b)** H7Y, **(c)** D9H, **(d)** D9N, **(e)** S22R, **(f)** N70T, **(g)** R75W, **(h)** G79R binding to dsDNA.
